# Evaluation of p53, Caspase-3, Bcl-2, and Ki-67 markers in oral squamous cell carcinoma and premalignant epithelium in a sample from Alava Province (Spain)

**DOI:** 10.4317/medoral.18901

**Published:** 2013-05-31

**Authors:** Antonio Bascones-Martínez, Carlos Rodríguez-Gutierrez, Enrique Rodríguez-Gómez, José A. Gil-Montoya, Rafael Gómez-Font, Miguel Á. González-Moles

**Affiliations:** 1 Oral Medicine and Periodontology, Department of Medicine and Bucofacial Surgery, Dental School, Complutense University of Madrid, Plaza Ramón y Cajal 3, Ciudad Universitaria, 28040-Madrid, Spain; 2 Department of Medicine and Bucofacial Surgery, Dental School, Complutense University of Madrid, Plaza Ramón y Cajal 3, Ciudad Universitaria, 28040-Madrid, Spain; 3 Hospital de Txagorritxu (Osakidetza) Alava Street, nº 45, 01006 Vitoria, Álava, Spain; 4 Professor, Department of Oral Medicine, School of Dentistry, Granada University, Campus de la Cartuja, Avda. de Madrid s/n, 18071-Granada, Spain; 5 Associate Professor, Department of Medicine and Bucofacial Surgery, Dental School, Complutense University of Madrid, Plaza Ramón y Cajal 3, Ciudad Universitaria, 28040-Madrid, Spain; 6 Department of Oral Medicine, School of Dentistry, Granada University, Campus de la Cartuja, Avda. de Madrid s/n, 18071-Granada, Spain

## Abstract

Objectives: The objective of this study was to determine whether alterations in the expression of p53, caspase-3 Bcl-2, and ki-67 appear early in premalignant oral epithelium and show clonal behavior. Study Design: Samples from 41 tumors with their adjacent non-tumor epithelia were immunohistochemically analyzed using monoclonal antibodies that recognize p53, caspase-3, Bcl-2, and Ki-67 Results: A statistically significant association was found between the expression in tumor and adjacent epithelium of p53, caspase-3, and Bcl-2 but not of k-67. A significant association was observed between the expression of ki-67 and p53 in both localizations. In non-tumor (premalignant) epithelium samples, there was a significant inverse relationship between the expressions of p53 and caspase-3 and a significant direct relationship between the expressions of p53 and Bcl-2. Conclusions: Alterations in these proteins appear to operate in combination with premalignant epithelia to create hyperproliferative cell states that favor the acquisition of summative oncogenic errors that confer invasive capacity.

** Key words:**Cell cycle, apoptosis, p53, caspase-3, Bcl-2, Ki-67.

## Introduction

Cancerous cells are able to elude growth-inhibiting signals and develop mechanisms to evade apoptosis (1). They enter a pathological proliferative process that does not respond to exogenous growth signals, losing the main cell proliferation control mechanisms linked to the expression of tumor suppressor genes, whose main function is to halt the cell cycle for DNA repair, and developing anti-apoptotic molecular mechanisms (2). These molecular alterations are essential for cancer development and for the persistence of an uncontrolled proliferative state, in which the probability of oncogenic errors related to cell division increases due to so-called genomic instability. It is currently accepted that the development of oral cavity cancer is attributable to summative oncogenic alterations that appear very early and result in a progression from epithelial dysplasia through in situ carcinoma to invasive disease (3,4). In this multistep process, precancerous epithelium may undergo malignant transformation through the alteration of chromosomal loci that host highly important tumor suppressor genes, such as 17p (5-7). Furthermore, molecular changes contributing to tumor development may be clonally transmitted by cells to their descendants, generating cell hierarchies predisposed to acquire new disorders that would eventually be responsible for persistent tumor growth.

Increasing knowledge of the molecular mechanisms involved in cancer development has enabled the development of molecular treatment strategies to reestablish the normal function of tumor suppressor genes in the tumor or to interrupt the intracellular pathways that transcribe aberrant growth signals, among other goals (1). Given that some disorders are established early, the application of these treatment strategies in premalignant cancer stages would help to prevent progression to invasion. This approach requires precise knowledge of early molecular events in the development of oral cancer (8).

Non-tumor epithelium adjacent to oral cancer represents a good example of a premalignant field in which antitumor control mechanisms have failed (9,10). In the present study, tumor and adjacent non-tumor epithelium (ANTE) samples were immunohistochemically analyzed, evaluating the expression of the following markers: ki-67, to determine the global fraction of proliferative cells as indirect evidence of self-sufficient tumor cell growth; p53, the main tumor suppressor protein; caspase 3, apoptotic marker; and Bcl-2, anti-apoptotic marker. The main objective of this study was to determine whether these proteins are altered early in premalignant fields and whether they behave clonally in cancer development.

## Material and Methods

The study included 41 patients with Oral Squamous Cell Carcinoma (OSCC) under treatment in the Hospital of Txagorritxu (Osakidetza-Vitoria-Spain). After approval of the study by the hospital ethics committee, the hospital records of patients were reviewed and data were gathered on age, sex, reason for the first hospital visit, and the clinicopathological characteristics of the lesions, including their localization, size, stage, and degree of differentiation. Clinicopathological variables were considered missing when not found in the clinical records. Formalin-fixed and paraffin-embedded tumor tissue was available for all patients. The presence of ANTE with the tumors was recorded.

All biopsies were incisional and performed before the treatment, which was tumor resection in all cases. Only four patients (15% of the sample) were treated with radiotherapy, and no patient received chemotherapy. According to the TNM staging system, 20 patients (50%) were T1N0M0, 17 patients (43%) T2N0M0, and 4 patients (7%) T3N0M0.

-Immunohistochemistry: For the immunohistochemical staining, 4-?m sections were cut from paraffin blocks. Peroxides–antiperoxidase and avidin–biotin techniques were applied. The study was performed automatically using Autostainer Link equipment (Dako, Carpintería, CA, USA) and EmVision™FLEX reagents (K8002; Dako, Carpintería, CA, USA). Primary anti-bodies were DO-7, Mib-1, anti-Bcl-2 (Dako, Carpintería, CA, USA) and anti-caspase-3 (Pharmagen, San Diego, CA). Manufacturers’ instructions were rigorously followed. For the negative control, the primary antibody was replaced with phosphate buffer saline. For the positive control, tissue was used from an OSCC sample known to intensively express each protein. The result was considered positive when a brown color appeared in the cell nucleus (p53 and ki-67) or cytoplasm (caspase-3 and Bcl.2). Expression in premalignant and malignant epithelium was assessed in four randomized high-power fields (40×). Total cell number and number of positive cells were counted in each field, obtaining a mean percentage expression in each case. The count was made from the image on the computer screen.

Samples were assigned to one of the following categories, according to the mean percentage expression of positive cells found in each case: 0% positive cells (?), 1–25% positive cells (+), 26–50% positive cells (++), 51–75% positive cells (+++), or >75% positive cells (++++). This categorization was applied for all antibodies. In order to simplify the immunohistochemistry study, the results were grouped in two categories (0-25% and 26-100%).

Histological and immunohistochemical analyses were always performed by the same experienced examiner (MAGM), who was blind to the clinical stage, treatment, and course of the disease. Furthermore, both intra- (MAGM vs MAGM, 1 week apart) and inter-examiner (MAGM vs JGM, another experienced examiner) concordances were tested by repeating the marker observations in 13 samples. Quadratic weighted kappa values for intra- and inter-examiner concordance ranged from 0.62 to 0.95, considered adequate according to the Landis & Koch scale (11).

-Statistical analysis

SPSS 1.9 for Windows (IBM, Chicago IL) was used for the data analyses. A descriptive statistical analysis was performed (FREQUENCIES procedure), expressing qualitative data as frequencies and rates. Contingency tables were constructed (CROSSTABS procedure) to determine relationships among qualitative variables. The chi-square test and Pearson chi-square test were used to analyze independent qualitative variables.

## Results

**Figure 1:**
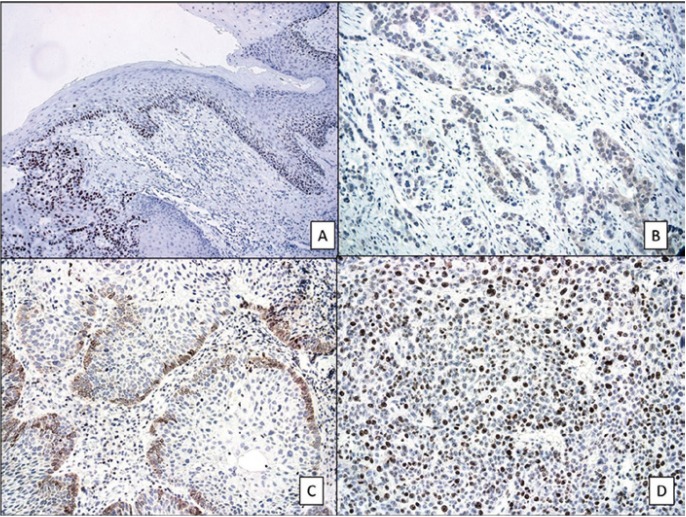
A) P53 nuclear expression in premalignant and malignant oral epithelia (x100; immunohistochemical technique). B) Cytoplasmatic expression of caspase-3 in oral carcinoma (x100; immunohistochemical technique). C) Cytoplasmatic expression of Bcl-2 at the invasion front of the oral carcinoma (x100; immunohistochemical technique). D) High expression of ki-67 in an oral carcinoma (x100; immunohistochemical technique).

 Table 1 shows the results of comparing expressions of p53, caspase 3, Bcl-2 and Ki 67 between tumor tissue and the accompanying ANTE. For all markers, with the exception of ki-67, a higher expression in ANTE was accompanied by a higher expression in tumor tissue.  Table 2 compares the expression of p53 with the expressions of caspase-3, Bcl-2, and Ki-67 in the tumor. A higher expression of ki-67 was accompanied by a higher expression of p53.  Table 3 compares the expression of p53 with those of caspase-3, Bcl-2, and Ki-67 in the ANTE (Fig. 1).

**Table 1:**
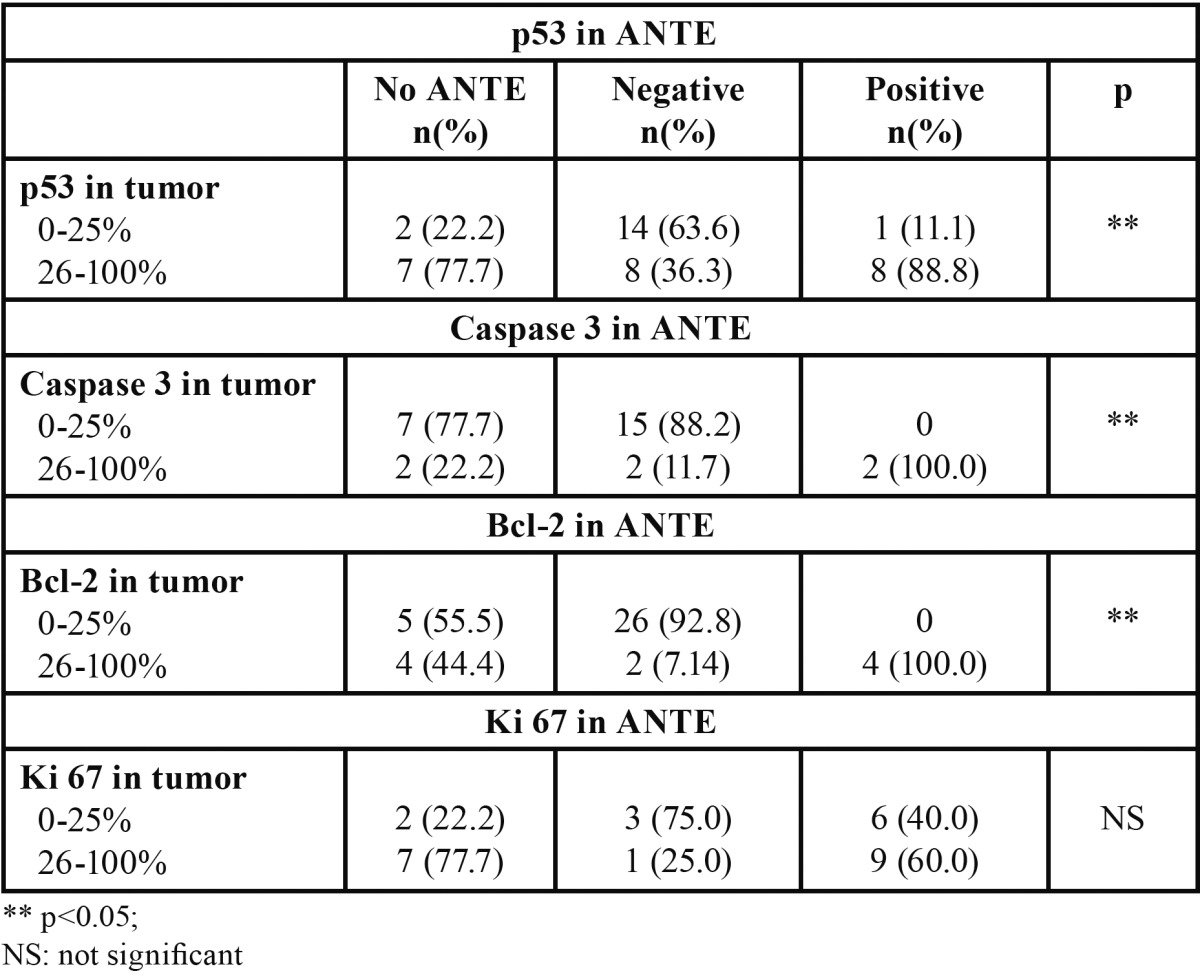
Comparison of expressions of p53, Caspase 3, and Bcl-2and Ki-67 between tumor epithelium and adjacent non-tumor epithelium (ANTE).

**Table 2:**
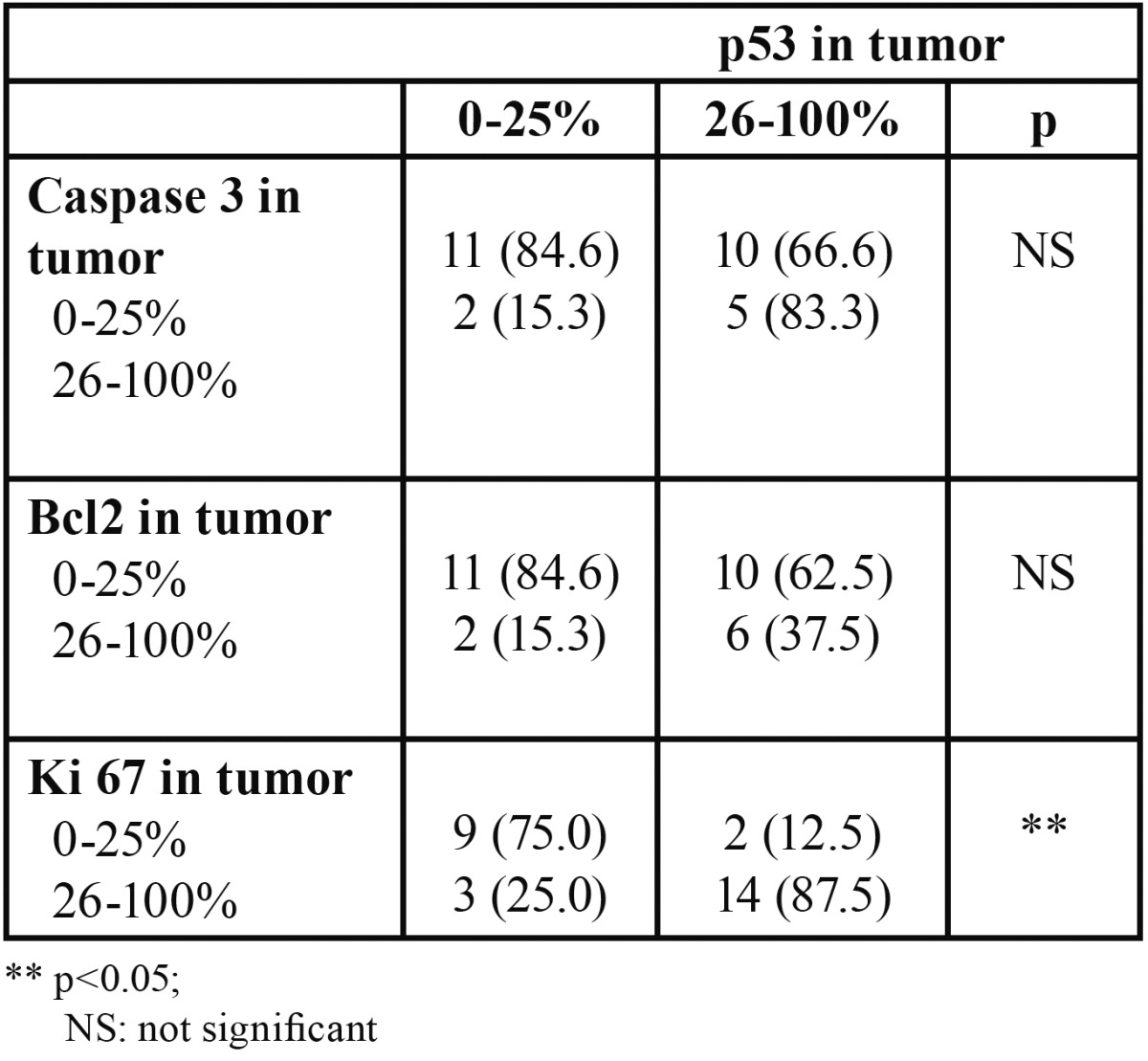
Association between expression of p53 and expressions of Caspase 3, Bcl-2, and Ki 67 in tumor samples.

**Table 3:**
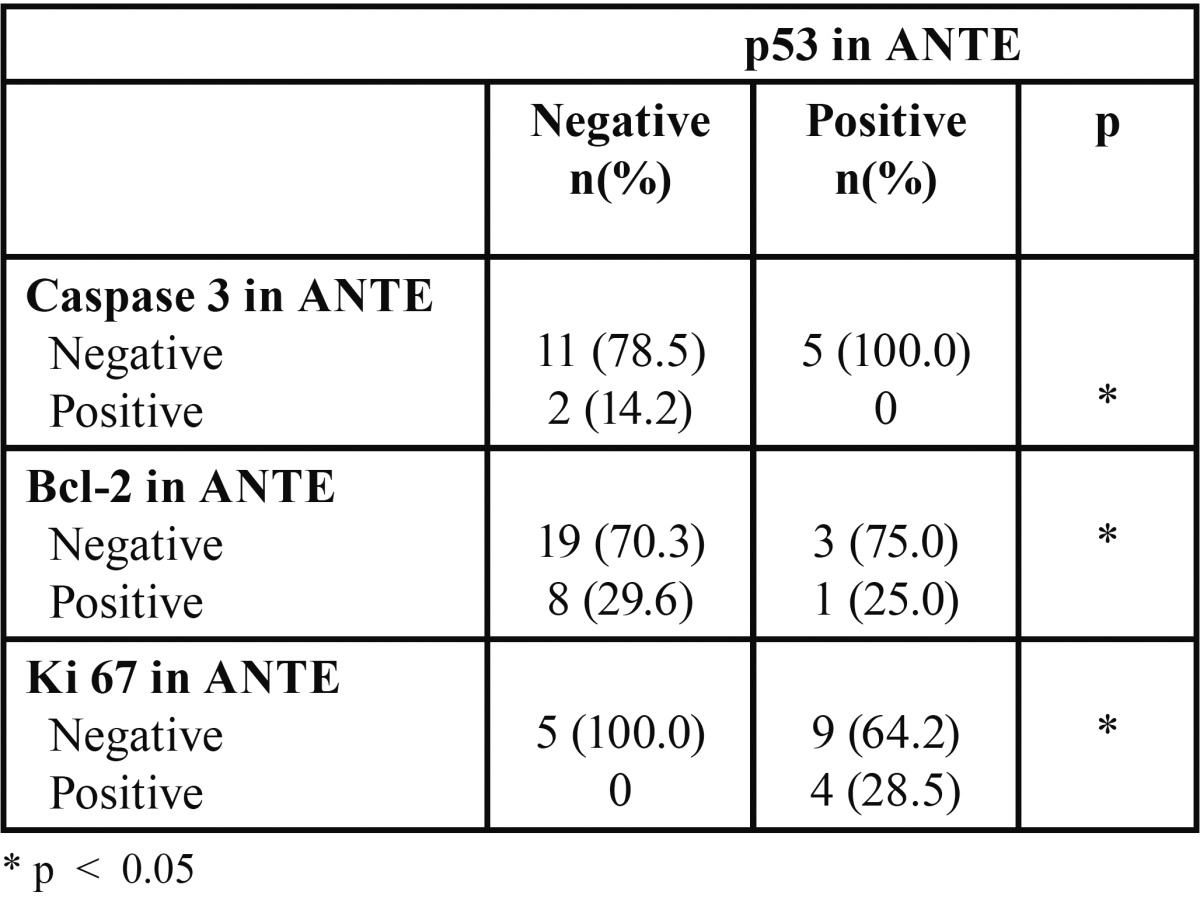
Association between expression of p53 and expressions of Caspase 3, Bcl-2, and Ki 67 in ANTE samples.

## Discussion

This study analyzed the expression of cell cycle regulators and proliferative markers in oral cancer. Limitations of this type of immunohistochemical study relate to the diverse antibodies used by different authors, the lack of agreement among researchers on cutoff points for considering a case positive, and difficulties in the evaluation. Nevertheless, immunohistochemical analysis yields considerable data on the topographical expression of markers and the relationships of positive cells with the surrounding cells. Our findings indicate that alterations in the expression and function of the main cell cycle-regulating proteins operate in combination with the multistep process of oral carcinogenesis to create a state of cell hyperproliferation (12), thereby favoring the occurrence of summative oncogenic events that enhance the proliferative and invasive activity of tumor cell clones. In this study, p53 expressions in OSCCs and in their associated ANTE were significantly associated ( Table 1), demonstrating that p53 alterations are clonal and early oncogenic events in the multistep carcinogenesis of the oral mucosa. Alterations of p53 constitute an essential component of the oncogenic alterations that create genomic instability and decisively contribute to the transformation process rather than being secondary to the genomic instability of cell clones in the malignancy pathway (13,14). Although immunohistochemistry has been reported to be an imprecise method to assess the mutational status of the p53 gene, due to discrepancies between protein expression and sequencing results, it is acknowledged that the immunohistochemical overexpression of p53 in premalignant epithelium indicates an incorrect functioning of the p53-related control system (5).

This interpretation is supported by our comparison between the expression of p53 and that of the other markers ( Table 1). Thus, we observed a significant association between p53 and ki-67 in both tumor and ANTE, which may indicate that the overexpression of p53 corresponds to the loss of its cell-cycle repression functions ( Table 2, Table 3). Moreover, our finding of an inverse association between the expression of p53 and that of caspase-3 suggests that p53 is unable to arrest the cell cycle and also loses its apoptosis-inducing function in these epithelia ( Table 2, Table 3). According to our results, the lack of apoptotic response in the ANTE is not only attributable to the loss of p53 function but also to a direct anti-apoptotic mechanism linked to the overexpression of Bcl-2 (15). The two oncogenic mechanisms (loss of p53 function and Bcl-2 overexpression) appear to operate in combination to suppress the apoptotic response in premalignant epithelium (16). Evidently, apoptosis suppression does not globally affect all of the cells of premalignant epithelia.

We observed caspase-3 expression in some areas of ANTE, and there was a significant association between the expression of caspase-3 in ANTE and tumor ( Table 3). In our opinion, this result indicates the presence of heterogeneous cell populations in premalignant fields and in the tumors that arise from them. Proliferation studies have shown that the persistent growth of a tumor or premalignant epithelium depends on a hierarchy of cells that have obtained a growth advantage, regardless of whether other cells in the same tissue develop apoptosis (9,10). Cells within the tumor that are capable of developing apoptosis would not con-tribute to persistent tumor growth.

Finally, the lack of association between the expression of ki-67 in ANTE and in the associated tumor ( Table 2, Table 3) indicates that the proliferative state in premalignant and malignant tissues varies according to the time course of the process and reflects the occurrence of late oncogenic events in the malignant cell population. These events would derive from genomic instability secondary to p53 changes, which would endow specific cell clones with proliferative and invasive advantages (17). These alterations would result from a loss of p53 function after gene mutation or from its inactivation by other causes and also from the effects of the demonstrated oncogenic functions of mutated p53 protein (2).

## Conclusions

Our results suggest that p53 alterations are decisive oncogenic events in multistep oral carcinogenesis, alongside other oncogenic molecular processes that prevent the apoptotic response and lead to malignant transformation. Immunohistochemistry is a simple procedure that can be routinely applied and may help to identify patients requiring special follow-up and therapeutic measures. However, a wider study is required to verify these conclusions.
